# Latent profile analysis for kinesiophobia in patients undergoing coronary artery bypass grafting

**DOI:** 10.3389/fresc.2026.1755424

**Published:** 2026-04-17

**Authors:** Qi Luo, Xiaojing Guo, Yi Xu, Yanqiu Yang, Mingzi Li

**Affiliations:** 1 Department of Nursing, Beijing Anzhen Hospital to Capital Medical University, Beijing, China; 2 School of Nursing, Peking University, Beijing, China

**Keywords:** coronary artery bypass, kinesiophobia, exercise rehabilitation, cardiac rehabilitation, cluster analysis

## Abstract

**Background:**

Kinesiophobia is a common psychological barrier among patients undergoing coronary artery bypass grafting (CABG), yet its heterogeneity remains unclear. Identifying distinct fear-of-movement patterns may support targeted rehabilitation.

**Methods:**

A cross-sectional study was conducted among patients scheduled for CABG (*N* = 296; 78.7% male; mean age=59.97 years, SD = 10.0). Latent profile analysis (LPA) was performed using the four domains of the Tampa Scale for Kinesiophobia Heart to identify subgroups. Model fit indices, entropy, and class interpretability guided the selection of the optimal model.

**Results:**

Three distinct latent profiles emerged, representing the best statistical fit and interpretability. The majority of participants exhibited moderate kinesiophobia (75.3%), followed by a low kinesiophobia group (22.3%). A small high kinesiophobia group (2.4%) demonstrated substantially elevated scores across all domains, especially in perceived danger and exercise avoidance.

**Conclusion:**

Three distinct latent profiles of kinesiophobia were identified among patients undergoing CABG, highlighting notable heterogeneity in fear-of-movement responses. These findings provide a basis for developing personalized rehabilitation strategies, and future studies should investigate the psychological and clinical factors underlying this heterogeneity and its implications for postoperative outcomes.

## 1 Introduction

Cardiovascular diseases remain the leading cause of death globally, accounting for approximately 31% of all deaths worldwide (1). Among these, coronary heart disease (CHD) is a leading condition requiring cardiovascular surgical interventions and poses a significant threat to public health. Although advances in prevention and treatment have reduced disease burden in many high-income countries, CHD prevalence continues to rise in numerous developing regions, creating substantial clinical and economic challenges (2). Current treatment strategies for CHD include pharmacological therapy, interventional procedures, and surgical interventions (3, 4). Among these, coronary artery bypass grafting (CABG) remains the preferred surgical option for complex coronary lesions and has demonstrated durable benefits in symptom relief and long-term survival (5).

Regular physical activity is a cornerstone of secondary prevention in cardiovascular care. Evidence consistently shows that exercise improves cardiopulmonary function, slows atherosclerotic progression, and reduces the risk of recurrent cardiovascular events (6). Nonetheless, adherence to recommended exercise regimens remains suboptimal in patients with heart disease, with approximately 70% failing to meet recommended activity levels within the first year post-treatment (7). Psychological barriers—particularly kinesiophobia, defined as an excessive and irrational fear of movement or physical activity due to perceived vulnerability or risk of harm—have been recognized as a critical factor undermining postoperative rehabilitation (8). Existing research indicates that heart disease patients commonly experience varying degrees of kinesiophobia both before and after surgery. A systematic review has reported that the prevalence of kinesiophobia among post-cardiac surgery patients ranges from 39.20% to 82.57%, and it can lead to functional decline, delayed recovery, and reduced engagement in physical activity, thereby further worsening overall health outcomes (9). For individuals undergoing CABG, concerns regarding surgical outcomes, sternal stability, and fear of provoking cardiac symptoms may further exacerbate avoidance behaviors (10).

Despite increasing recognition of the clinical importance of kinesiophobia, the majority of current studies rely on variable-centered methodologies such as regression or correlation analyses. These approaches assume population homogeneity and therefore may obscure important inter-individual differences. Given the psychological, clinical, and experiential diversity of patients preparing for CABG, substantial unobserved heterogeneity likely exists in how kinesiophobia manifests. A person-centered analytical framework, such as latent profile analysis (LPA), provides a more suitable approach for identifying distinct subgroups of patients who share similar fear-of-movement patterns (11). Characterizing these subtypes may offer valuable insights into differential risk profiles, psychological readiness for surgery, and potential targets for tailored intervention.

To date, however, no studies have applied a person-centered approach to delineate kinesiophobia profiles specifically among patients scheduled for CABG. Addressing this gap is crucial, as early identification of high-risk psychological patterns may enable clinicians to implement customized preoperative counseling and rehabilitation strategies, ultimately improving postoperative recovery and long-term physical activity engagement.

Therefore, the present study aims to employ latent profile analysis to identify distinct kinesiophobia subtypes among patients undergoing CABG and provide an empirical basis for personalized rehabilitation interventions. By uncovering the heterogeneity of fear-of-movement responses, this study may contribute to a better understanding of psychological readiness for cardiac surgery and inform the development of more patient-centered care pathways.

## 2 Materials and methods

### 2.1 Study design

This is an observational, cross-sectional study to examine the heterogeneity of kinesiophobia among patients undergoing CABG. The study was conducted in accordance with the Strengthening the Reporting of Observational Studies in Epidemiology (STROBE) guidelines for cross-sectional studies. The study protocol was approved by the Ethics Committee of Beijing Anzhen Hospital (approval number: 2026107x). Written informed consent was obtained from all participants prior to data collection to ensure voluntary participation.

### 2.2 Participants

A total of 296 hospitalized patients who were admitted for and underwent CABG surgery were consecutively recruited from a tertiary general hospital in Beijing, China, between October and November 2024.

The inclusion criteria were as follows: (1) Met the diagnostic criteria for CHD as outlined in the Guidelines for Primary Prevention of Cardiovascular Disease in China; (2) undergoing first-time CABG surgery with surgical indications; (3) Classified as New York Heart Association (NYHA) functional class ≤ III; Aged ≥ 18 years; (4) Provided informed consent to participate in the study.

Exclusion criteria were: (1) Congenital motor dysfunction; (2) Lack of verbal communication abilities or presence of psychiatric disorders; (3) Unstable acute cardiovascular events; (4) Severe dysfunction or significant impairment of the respiratory system, liver, or kidneys.

All 296 eligible patients completed the survey. No participants were excluded after recruitment, and no missing data were observed in the final dataset.

### 2.3 Variables and measures

A self-designed questionnaire was used to collect general information, which consisted of two sections: sociodemographic variables and disease-related variables. Sociodemographic variables included age, gender, body mass index (BMI), educational level, and marital status. Disease-related variables included disease duration, and comorbidities.

Kinesiophobia in this study was assessed using the Tampa Scale for Kinesiophobia Heart (TSK-SV Heart), a widely validated instrument for evaluating kinesiophobia in patients with cardiovascular disease (8). This 17-item scale evaluates four key dimensions: two behavior-focused constructs, ‘Avoidance of exercise’ (5 items) and ‘Dysfunctional self’ (4 items), reflecting irrational beliefs and avoidance behaviors related to rehabilitation; and two belief-oriented constructs, ‘Perceived danger for heart problem’ (4 items) and ‘Fear of injury’ (4 items), which capture patients’ perceptions of risk and fear associated with exercise (12). Each item is rated on a 4-point Likert scale, ranging from strongly disagree to strongly agree, with total scores ranging from 17 to 68. Higher scores indicate greater levels of kinesiophobia. In the present study, the internal consistency of the scale was acceptable, with a Cronbach's *α* of 0.773 for the total scale and ranging from 0.641 to 0.734 across the four subscales.

### 2.4 Statistical analysis

Statistical analyses were conducted using SPSS version 26.0 (IBM Corp., Armonk, NY, USA) and LPA in Mplus version 8.3. Descriptive statistics were performed in SPSS. Categorical variables were presented as frequencies and percentages, while continuous variables were summarized as means ± standard deviations (SDs).

LPA, a person-centered analytical approach, was employed to identify latent subgroups within the dataset by capturing individual heterogeneity based on continuous variables (13). Compared to traditional classification methods, LPA has demonstrated superior precision and more meaningful subgroup identification (11). In this study, each dimension of the TSK-SV Heart scale was used as a key variable in constructing the latent profile model. To determine the best-fitting model, multiple fit indices and statistical criteria were evaluated. The Lo–Mendell–Rubin (LMR) test and the bootstrap likelihood ratio test (BLRT) were used to compare models with k and k-1 classes, where a significant *p*-value supported the k-class model as a better fit. Model selection was further guided by entropy, the Akaike Information Criterion (AIC), the Bayesian Information Criterion (BIC), the adjusted Bayesian Information Criterion (aBIC), and the interpretability of the latent classes (14). Lower AIC, BIC, and aBIC values indicated improved model fit. Entropy values were used to assess the classification accuracy of each model, with higher values indicating more accurate categorization (ideally above 0.80). To support the interpretation of the chosen solution, z-scores with a mean 0 and standard deviation 1 were calculated and used.

## 3 Results

### 3.1 Patients’ characteristics

A total of 296 adults were included in this study. Participants’ ages ranged from 39 to 87 years, with a mean of 59.97 years (SD = 10.0). The majority were male (78.7%). In terms of education level, 55.1% had completed middle school or below. 94.3% of the participants were married, and 87.5% had at least one coexisting chronic disease. Detailed demographic and clinical characteristics are presented in Table 1.

**Table 1:** Participant characteristics.

Variable	All (*n* = 296)	Low (*n* = 66)	Moderate (*n* = 223)	High (*n* = 7)
Age, mean (SD), years	59.97 (10.0)	60.53 (9.8)	59.85 (10.2)	58.7 (7.9)
Gender, *n* (%)				
Male	233 (78.7)	50 (75.8)	177 (79.4)	6 (85.7)
Female	63 (21.3)	16 (24.2)	46 (20.6)	1 (14.3)
Body mass index, mean (SD), kg/m^2^	25.91 (4.0)	25.30 (2.6)	26.13 (4.3)	24.63 (4.9)
18.5∼24.9, *n* (%)	127 (42.9)	29 (43.9)	93 (41.7)	5 (71.4)
≥25, *n* (%)	169 (57.1)	37 (56.1)	130 (58.3)	2 (28.6)
Education level, *n* (%)				
Middle school or below	163 (55.1)	37 (56.1)	124 (55.6)	2 (28.9)
High school or vocational	105 (35.5)	21 (31.8)	81 (36.3)	3 (42.9)
College or above	28 (9.5)	8 (12.1)	18 (8.1)	2 (28.6)
Marital status				
Married	279 (94.3)	61 (92.4)	211 (94.6)	7 (100.0)
Single/Divorced/Widowed	17 (5.7)	5 (7.6)	12 (5.4)	0 (0.0)
Disease duration, mean (SD), years	4.24 (5.6)	3.48 (5.3)	4.44 (5.6)	5.14 (8.2)
≤1 year, *n* (%)	157 (53.0)	40 (60.6)	113 (50.7)	4 (57.1)
>1–5 years, *n* (%)	59 (19.9)	12 (18.2)	46 (20.6)	1 (14.3)
>5 years, *n* (%)	80 (27.0)	14 (21.2)	64 (28.7)	2 (28.6)
Combined with other chronic diseases				
No, *n* (%)	37 (12.5)	10 (15.2)	27 (12.1)	0 (0.0)
Yes, *n* (%)	259 (87.5)	56 (84.8)	196 (87.9)	7 (100.0)
Smoking status				
No, *n* (%)	140 (47.3)	37 (56.1)	101 (45.3)	2 (28.6)
Yes, *n* (%)	156 (52.7)	29 (43.9)	122 (54.7)	5 (71.4)
Alcohol consumption				
No, *n* (%)	189 (63.9)	43 (65.4)	142 (63.7)	4 (57.1)
Yes, *n* (%)	107 (36.1)	23 (34.8)	81 (36.3)	3 (42.9)
Kinesiophobia scores, mean (SD)	39.03 (4.8)	33.52 (2.4)	40.22 (3.4)	53.29 (3.6)
Fear of injury	10.10 (1.7)	8.15 (1.4)	10.57 (1.2)	13.57 (2.2)
Perceived danger for heart problem	8.23 (1.4)	7.53 (1.3)	8.32 (1.3)	11.86 (1.8)
Avoidance of exercise	11.06 (1.6)	10.09 (1.3)	11.24 (1.4)	14.57 (1.0)
Dysfunctional self	9.74 (1.6)	7.74 (1.2)	10.22 (1.1)	13.29 (0.8)

### 3.2 Potential profiles of kinesiophobia

LPA was conducted to examine the heterogeneity of kinesiophobia among patients undergoing CABG. Models with one to five profiles were evaluated, and the corresponding model fit indices are summarized in Table 2. As the number of classes increased, the AIC, BIC, and aBIC values demonstrated a consistent decline, indicating progressive improvement in model fit. The three-class solution was selected as the optimal model based on a combination of statistical criteria and interpretability. Specifically, this model demonstrated relatively lower information criterion values, the highest entropy (0.839), and statistically significant results for both the LMR and BLRT, suggesting a better fit compared with the two-class model. In addition, the three-class solution provided greater differentiation of kinesiophobia profiles. In contrast, although models with four or more classes showed slightly lower information criterion values, the LMR and BLRT results were no longer statistically significant, suggesting that additional classes did not provide a meaningful improvement in model fit.

**Table 2:** Model fit indices for latent profile models with different numbers of classes (*N* = 296).

Models	AIC	BIC	aBIC	Entropy	*P* value		Estimated class proportions
					LMR	BLRT	
1	4,433.554	4,463.076	4,437.706	-	-	-	-
2	4,324.521	4,372.495	4,331.268	0.624	0.1489	<0.0001	0.334/0.666
**3**	**4,234**.**572**	**4,300**.**998**	**4,243**.**914**	**0**.**839**	**0**.**0170**	**0**.**0154**	**0.753/0.223/0.024**
4	4,212.324	4,297.202	4,224.262	0.796	0.1218	0.1132	0.291/0.635/0.051/0.024
5	4,203.988	4,307.318	4,218.521	0.826	0.0950	0.0909	0.294/0.632/0.047/0.017/0.010

The three-class latent profile model was identified as the optimal solution. Compared with models with fewer classes, it exhibited lower information criterion values, indicating better model fit, and the highest entropy (0.839), reflecting strong classification accuracy. Both the LMR (*p* = 0.017) and the BLRT (*p* = 0.0154) were statistically significant, further supporting the superiority of the three-class solution over the two-class model. The estimated class proportions (0.753, 0.223, 0.024) reflect the relative prevalence of each kinesiophobia profile among patients undergoing CABG.

AIC, Akaike information criterion; BIC, Bayesian information criterion; aBIC, adjusted Bayesian information criterion; LMR, Lo–Mendell–Rubin likelihood ratio test; BLRT, bootstrap likelihood ratio test.

However, it should be noted that the smallest class in the three-class solution accounted for only 2.4% of the sample, which may raise concerns regarding the stability and generalizability of this subgroup. Therefore, while the three-class model was retained due to its overall statistical performance and interpretability, the findings related to this small class should be interpreted with caution. Overall, these profiles reflect relative differences in kinesiophobia within the sample.

**Figure 1:**
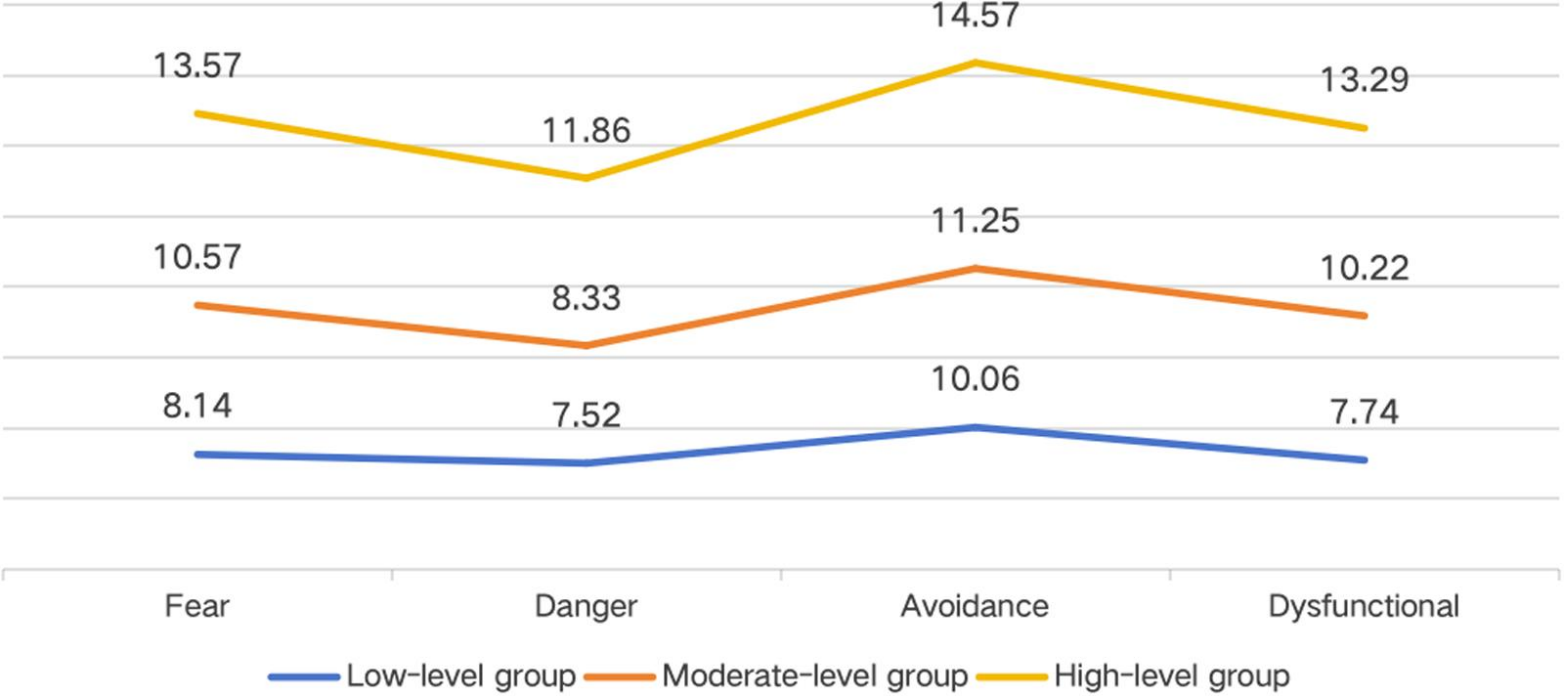
The scores of the three potential categories of patients with coronary heart disease. Fear, Fear of injury; Danger, Perceived danger for heart problem; Avoidance: Avoidance of exercise; Dysfunctional, Dysfunctional self.

**Figure 2:**
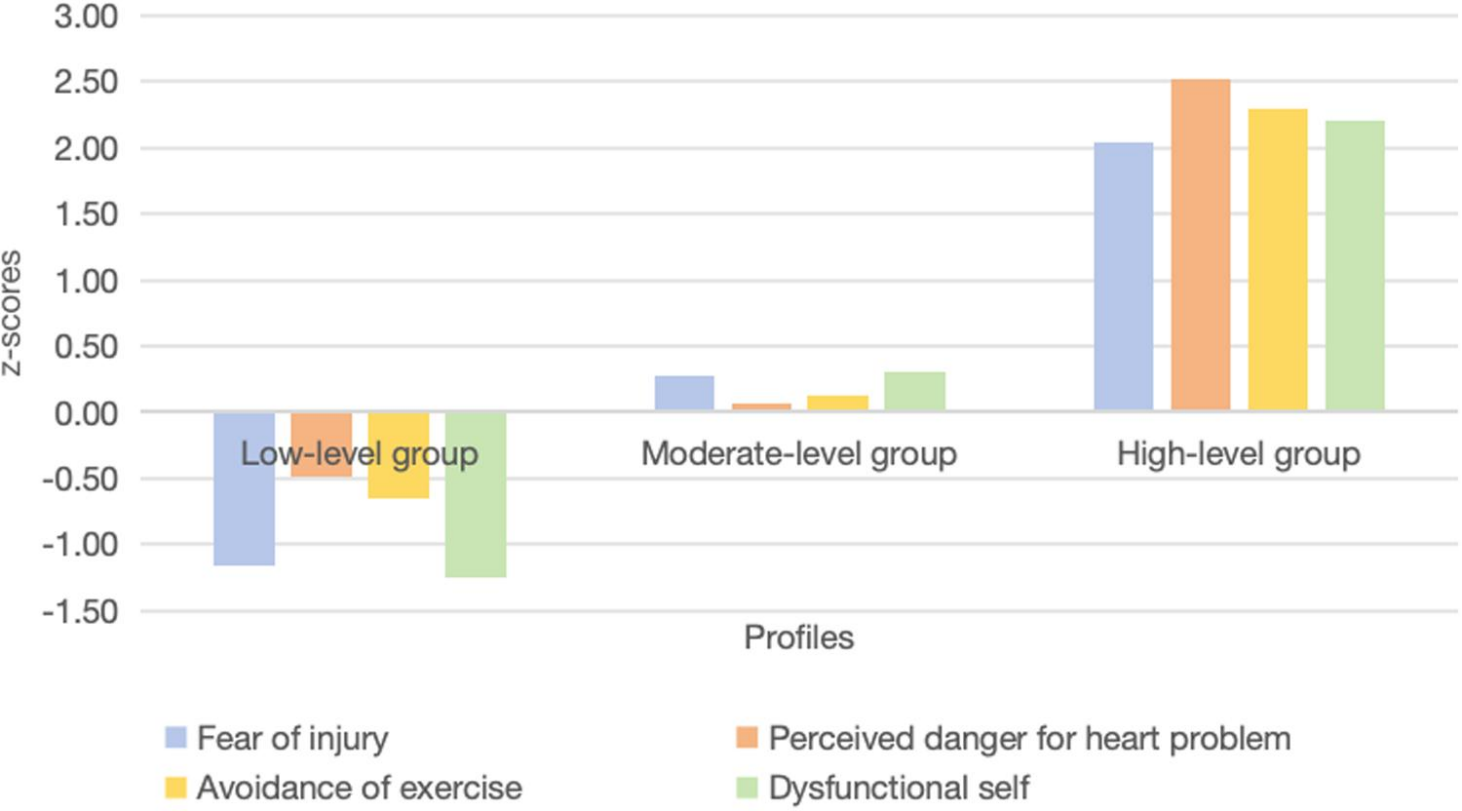
Graphs of profiles based on Tampa Scale for Kinesiophobia Heart scale four domains.

The nomenclature for latent profiles was determined by examining the response probability patterns across items within each profile and comparing the mean values of grouping variables across the latent classes. The total scores of the three identified latent categories across the four domains are depicted in Figure 1. To facilitate interpretation, z-scores (mean = 0, standard deviation = 1) were computed; the resulting standardized scores are illustrated in Figure 2.

#### 3.2.1 Profile 1: “low kinesiophobia”

The first profile comprised 22.3% (*n* = 66) of the sample and was characterized by relatively lower scores across all four domains of kinesiophobia. All z-score values were lower than the mean of the whole sample for each of the domains considered. Specifically, lower values were observed in the ‘Fear of injury’ (z = −1.15) and ‘Dysfunctional self’ (z = −1.25) domains, as well as in the ‘Perceived danger for heart problem’ (z = −0.49) and ‘Avoidance of exercise’ (z = −0.65) domains.

#### 3.2.2 Profile 2: “moderate kinesiophobia”

The second profile was the largest, comprising 75.3% (*n* = 223) of the sample. It was characterized by scores close to the sample mean across all four domains, with z-scores ranging from −0.08 to +0.30. Patients reported a degree of kinesiophobia that was approximately average in the ‘Fear of injury’ (z = 0.28), ‘Perceived danger for heart problem’ (z = 0.07), ‘Avoidance of exercise’ (z = 0.12), and ‘Dysfunctional self’ (z = 0.30) domains.

#### 3.2.3 Profile 3: “high kinesiophobia”

The third profile was the smallest, comprising 2.4% (*n* = 7) of the sample. This profile was characterized by relatively higher scores across all domains, with z-scores exceeding 2.0 standard deviations above the sample mean in most domains. The highest values were observed for ‘Perceived danger for heart problem’ (z =  + 2.52) domain, followed by ‘Avoidance of exercise’ (z = 2.30), ‘Dysfunctional self’ (z = 2.21), and ‘Fear of injury’ (z = 2.04).

## 4 Discussion

This study employed a person-centered analytic approach to examine heterogeneity in kinesiophobia among patients undergoing CABG. Using LPA, three distinct subgroups were identified—low, moderate, and high kinesiophobia—demonstrating that fear-of-movement responses are not uniform in this clinical population but instead characterized by meaningful psychological variability.

The present findings align with prior studies that have similarly identified multiple kinesiophobia subtypes in cardiac populations. For example, Wang et al. conducted an LPA among patients with CHD and also reported three subgroups—low, moderate, and high kinesiophobia (1). However, our study extends this line of research by focusing on patients scheduled for CABG, a population that may present with more advanced disease and greater symptom burden. It is possible that such clinical characteristics, along with unmeasured factors such as preoperative anxiety, could contribute to heightened fear-related cognitions and avoidance tendencies. However, these variables were not directly assessed in the present study and should therefore be interpreted with caution. Furthermore, unlike previous item-level LPA approaches, our study adopted a domain-based profiling strategy, examining four distinct dimensions of kinesiophobia—fear of injury, perceived danger for heart problems, avoidance of exercise, and dysfunctional self. This approach provides a more theoretically grounded and clinically interpretable differentiation of patients, revealing nuanced patterns of kinesiophobia that may not be captured by item-level analyses.

The moderate kinesiophobia group constituted the majority (75.3%), followed by the low kinesiophobia group (22.3%). Both subgroups demonstrated relatively higher z-scores on fear of injury and dysfunctional self compared with the danger and avoidance domains. Several mechanisms may explain this pattern: (1) High organic symptom awareness before CABG. Patients with CHD often experience chest discomfort, dyspnea, or fatigue during activity, which can heighten fear of bodily harm even when their overall kinesiophobia level is low or moderate (15). (2) Concerns about physical frailty and self-efficacy. “Dysfunctional self” reflects doubts regarding physical capacity and recovery ability. Many CABG candidates perceive themselves as vulnerable due to long-standing illness, which may persist regardless of their broader emotional profile (16). These findings suggest that even patients with relatively low or moderate overall kinesiophobia retain domain-specific vulnerabilities that could hinder postoperative rehabilitation if not addressed.

Although representing only 2.4% of the sample, the high kinesiophobia group demonstrated clinically significant elevations across all domains, with z-scores exceeding +2 SD. Notably, this subgroup exhibited disproportionally high scores in perceived danger for heart problems and avoidance of exercise. Several factors may contribute to these exaggerated perceptions: (1) Intensified preoperative anxiety. CABG is a major surgical procedure, and some patients experience pronounced fear of surgical risks, graft failure, or postoperative complications, which may manifest as heightened cardiac danger beliefs (17). (2) Prior adverse experiences or misinformation. Patients may have experienced abrupt symptom exacerbations during activity or been cautioned excessively by family members or healthcare providers, reinforcing avoidance (18). Given the strong association between kinesiophobia, reduced physical activity, delayed functional recovery, and poorer long-term cardiac outcomes, individuals in this high-risk profile warrant targeted and early intervention.

### 4.1 Clinical implications

The identification of three distinct kinesiophobia profiles carries meaningful clinical value. First, the demonstrated heterogeneity highlights the need for personalized preoperative psychological assessment. Screening for kinesiophobia should be incorporated into routine preoperative evaluation to identify high-risk patients early. Second, interventions should be tailored to profile-specific needs. Low and moderate groups may benefit most from focused education, reassurance regarding sternal and cardiac safety during activity, and structured goal-setting to strengthen exercise self-efficacy. High kinesiophobia patients require more intensive strategies, such as cognitive restructuring, gradual exposure plans, anxiety management, and multidisciplinary counseling to address maladaptive danger beliefs and avoidance. Third, integrating kinesiophobia assessment into cardiac rehabilitation planning could enhance adherence, functional recovery, and long-term cardiac protection.

### 4.2 Limitations

Several limitations should be acknowledged. First, the single-center design and modest sample size—particularly the very small high-kinesiophobia subgroup—may limit generalizability and reduce the statistical stability of small-class estimates. Larger, multicenter studies are needed to validate the identified profiles and explore their determinants across diverse sociodemographic strata. Second, the reliance on self-reported data introduces the potential for recall bias, social desirability bias, and symptom misinterpretation. Future research should incorporate objective measures of activity, functional capacity, and psychological distress. Third, longitudinal studies are needed to examine how profile membership influences postoperative recovery trajectories and long-term rehabilitation outcomes.

## 5 Conclusion

This study identified three distinct latent profiles of kinesiophobia among patients undergoing CABG, highlighting significant heterogeneity in fear-of-movement responses. These findings provide an empirical basis for understanding individual differences in kinesiophobia and may inform the development of personalized rehabilitation and management strategies. Further research is needed to explore the clinical implications of these profiles for rehabilitation outcomes and to identify the underlying factors contributing to this heterogeneity.

## Data Availability

The original contributions presented in the study are included in the article, further inquiries can be directed to the corresponding author.
